# Artificial Magnetic Pattern Arrays Probed by Polarized Neutron Reflectivity

**DOI:** 10.3390/nano10050851

**Published:** 2020-04-28

**Authors:** Dmitry Gorkov, Boris P. Toperverg, Hartmut Zabel

**Affiliations:** 1 II. Physikalisches Institut, Universität zu Köln, 50937 Köln, Germany; 2 Festkörperphysik/Experimentalphysik, Ruhr-Universität Bochum, 44780 Bochum, Germany; 3 Petersburg Nuclear Physics Institute, NRC Kurchatov Institute, 188300 Gatchina, Russia

**Keywords:** neutron reflectometry, magnetic nanostructures, magnetic multilayers, polarization analysis, coherence volume, 75.70.Cn

## Abstract

Traditionally, neutron scattering is an essential method for the analysis of spin structures and spin excitations in bulk materials. Over the last 30 years, polarized neutron scattering in terms of reflectometry has also contributed largely to the analysis of magnetic thin films and magnetic multilayers. More recently it has been shown that polarized neutron reflectivity is, in addition, a suitable tool for the study of thin films laterally patterned with magnetic stripes or islands. We provide a brief overview of the fundamental properties of polarized neutron reflectivity, considering different domain states, domain fluctuations, and different domain sizes with respect to the neutron coherence volume. The discussion is exemplified by a set of simulated reflectivities assuming either complete polarization and polarization analysis, or a reduced form of polarized neutron reflectivity without polarization analysis. Furthermore, we emphasize the importance of the neutron coherence volume for the interpretation of specular and off-specular intensity maps, in particular when studying laterally non-homogeneous magnetic films. Finally, experimental results, fits, and simulations are shown for specular and off-specular scattering from a magnetic film that has been lithographically patterned into a periodic stripe array. These experiments demonstrate the different and mutually complementary information that can be gained when orienting the stripe array parallel or perpendicular to the scattering plane.

## 1. Introduction

Nanomagnetism is a key field for fundamental studies and technical applications of magnetic materials on the nanometer scale. In particular, in spintronics and magnonics, nanomagnetic systems are widely used as sensors, for logic circuits, and for data storage [1,2,3,4]. Nanomagnetic systems either consist of artificially arranged multilayers or laterally patterned islands, or a combination thereof. The artificial arrangements of various magnetic elements or compounds generates a rich environment for studying the effect of interfaces, symmetries, and interactions on their magnetic properties. Among the many methods that are used for analyzing the magnetism of artificial magnetic nanostructures, neutron scattering is less common but very powerful. Neutron scattering is more prominent for probing the magnetic properties of bulk materials. However it can also be highly useful as an analytical tool for studying magnetic nanostructures. In particular, neutron reflectivity provides detailed information on the mean magnetization profile perpendicular to the layers, on the reversal mechanism at coercivity, and on the domain distribution throughout the magnetic hysteresis.

The main aim of this contribution is a discussion of polarized neutron reflectivity (PNR) on flat magnetic thin films as well as off-specular scattering (OSS) on thin films patterned into a periodic array of micro-stripes. We start by recalling some basic properties of neutron reflectivity on flat magnetic films. Some earlier reviews of the theoretical background of PNR can be found, for instance, in Ref. [5,6]. Moreover, we highlight the importance of the coherence volume [7] when performing neutron scattering experiments on artificially patterned nanostructures [8,9]. These considerations are exemplified by experimentally determined neutron specular reflectivity and off-specular scattering results on a magnetic stripe array [10,11,12,13,14]. Neutron specular reflectivity on non-magnetic stripe arrays (gratings) has been discussed earlier by Majkrzak and coworkers [15], also emphasizing the importance of neutron coherence. Our focus here is on magnetic stripe patterns oriented parallel and perpendicular to the scattering plane.

## 2. Remarks on Neutron Specular Reflection and Off-Specular Scattering

### 2.1. Specular Reflection

**Figure 1:**
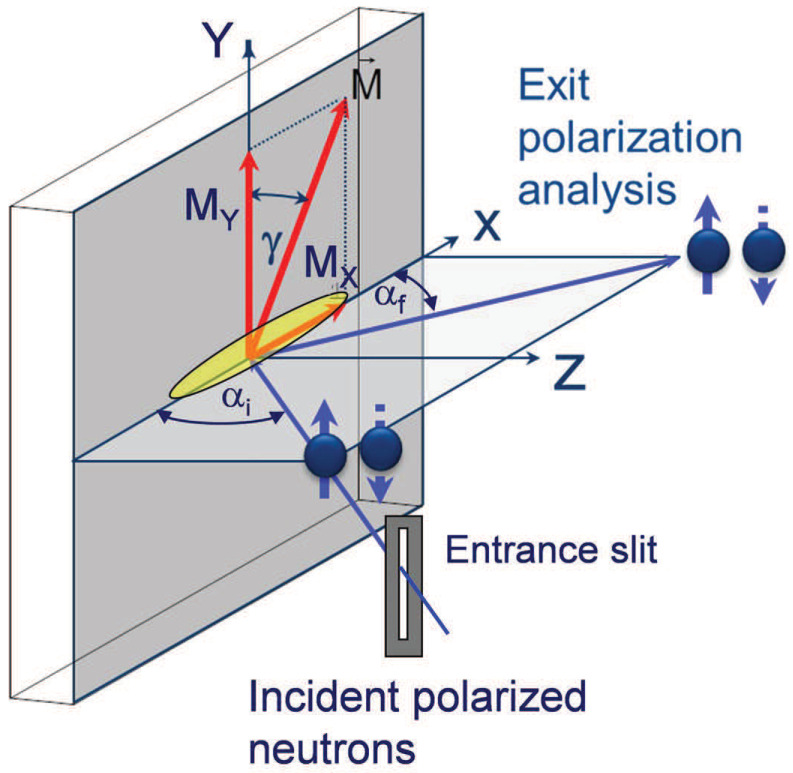
Sketch of the sample and beam geometry for polarized neutron reflectometry (PNR) experiments with one-dimensional polarization analysis along the *Y*-axis. The elongated ellipsoid indicates the coherence volume of neutrons defined by the beam divergence and the wavelength spread.

In the most common experimental geometry shown in Figure 1, the polarization vector of the incident neutron beam is fixed along with, or opposite to the a guide field B parallel to the *Y*-direction and perpendicular to the X,Z-scattering plane. Hence the guiding field serves as the quantization axis for the neutron spin whose projection onto this axis may either remain unchanged, or flip during the reflection process. The flat sample is assumed to be parallel to the X,Y-plane with the surface normal parallel to the *Z*-direction.

With this set-up, polarized neutron reflectometry allows to determine four specular reflectivities $R^{\pm ,\mp }\left(Q\right)$ as a function of the scattering vector *Q* by flipping the direction of the polarization of the incident beam and by analyzing the polarization of the reflected beam. Here *Q* is the modulus of the scattering vector Q defined by Q=(4π/λ)sinα, where λ is the neutron wavelength and α is the glancing incident (exit) angle of the neutron beam. In case of specular reflectivity, $Q\;=\;Q_{z}$ is oriented parallel to the film normal or perpendicular to the film plane. In general, $R^{\pm ,\mp }\left(Q_{z}\right)$ is the Fourier transform of the respective nuclear and magnetic scattering length density profiles $Nb_{n}\left(z\right)$ and $Nb_{m}\left(z\right)$ in the *Z*-direction, and *N* is the nuclear number density. For a homogeneously magnetized sample with magnetization vector tilted on the angle γ with respect to the *Y*-axis and assuming ideal neutron polarization, the non-spin-flip (NSF) reflectivities are defined by the equations [7]:

(1) $$\begin{array}{c} R^{++}=\frac{1}{4}{\left|\left(r_{+}+r_{-}\right)+\left(r_{+}-r_{-}\right)\cos\gamma \right|}^{2},\\ R^{--}=\frac{1}{4}{\left|\left(r_{+}+r_{-}\right)-\left(r_{+}-r_{-}\right)\cos\gamma \right|}^{2}, \end{array}$$

while the spin-flip (SF) reflectivities are written as:

(2) $$\begin{array}{c} R^{+-}=R^{-+}=\frac{1}{4}{\left|r_{+}-r_{-}\right|}^{2}\sin^{2}\gamma . \end{array}$$

Here $r_{\pm }$ are the complex reflection amplitudes determined by the sum or the difference of nuclear and magnetic scattering length densities (SLDs): $Nb_{\pm }=Nb_{n}\pm Nb_{m}$, where $b_{n}$ is the coherent nuclear scattering length, and $b_{m}$ is an effective magnetic scattering length (mSLD). The latter is proportional to the magnetic induction *B* [16]:

(3) $$Nb_{m}=CB\;\;\;\;$$

where the constant *C* is expressed by

(4) $$C=\frac{m_{n}\mu_{n}}{2\pi \hbar^{2}}=2.31\times 10^{-4}{\mathrm{nm}}^{-2}T^{-1}$$

Here, $m_{n}$ is the neutron mass, $\mu_{n}=\gamma_{n}\mu_{N}$ is the neutron magnetic moment, where $\gamma_{n}$ = 1.913 is the gyromagnetic ratio of neutrons and $\mu_{N}$ is the nuclear magneton. The Equation (3) does not depend on the orientation of the sample magnetization vector M with respect to e.g., the direction of the neutron beam polarization.

In contrast, the reflectivities in Equations (1) and (2) depend on the angle γ between the incident polarization vector collinear with the *Y*-axis and the sample magnetization vector M. More specifically, the NSF reflectivities are determined by the projection $m_{y}=\cos\gamma$ of the unit vector m=M/M onto the *Y*-axis, while the SF reflectivities are due to the component $m_{x}=\sin\gamma$ orthogonal to the *Y*-axis.

From Equation (1) it follows that NSF reflectivities, $R^{++}=R^{--}$, are degenerated under the following conditions: (1) the sample is non-magnetic ($Nb_{m}$ = 0); (2) the sample is totally demagnetized so that its mean magnetization 〈M〉=0; (3) the magnetization vector is oriented parallel to the *X*-axis ($\gamma =\pm \;90^{\circ }$); (4) the magnetization vector is oriented parallel to the *Z*-axis (perpendicular anisotropy). In the latter case, the magnetic induction does not cause any reflection contrast at the surface of homogeneously magnetized film, and therefore PNR is not sensitive to the magnetization. If, alternatively, the magnetization vector 〈M〉≠0 and has a component $M_{Y}$ collinear with the *Y* axis, then $R^{++}\neq R^{--}$. In contrast, the SF-reflectivities are always equal to each other: $R^{+-}$ = $R^{-+}$. The case γ>0 is discussed further below.

In accordance with Equation (1), the difference of the NSF reflectivities is:

(5) $$R^{++}-R^{--}=\left(\right|r_{+}{|}^{2}-|r_{-}{|}^{2})\cos\gamma ,$$

while due to Equation (2) the SF reflectivities are proportional to $\sin^{2}\gamma$ with the proportionality coefficient $|r_{+}-r_{-}{|}^{2}/4$. Therefore, only the absolute value, but not the sign of the angle γ can be determined. Hence, with 1D polarization analysis it is impossible to determine the sense of the homogeneous magnetization rotation. This is usually not a problem when, for example, the film magnetization is tilted under the action of a magnetic field applied at an angle with respect to the uniaxial, or unidirectional anisotropy axis. On the other hand, homogeneous magnetization rotation is not always a unique mechanism of the magnetization evolution under the action of an external field. Often magnetization rotation competes with domain formation, which may become more favorable at certain field strengths and directions. These two scenarios of magnetization reversal processes can well be discriminated with PNR methods, being one of the main motivations for using PNR as a powerful analytical tool to analyze magnetism in nanostructures.

In case of domains, Equations (1)–(5) are also valid after averaging over the sample surface. Such averaging is easy to perform if the domains are sufficiently large so that each of them comprise a number of coherence ellipsoids. We call such domains “large”, in contrast to “small” domains, which fit into one single coherence ellipsoid. Coherence ellipsoids are discussed in the next Section 2.2. Thus, the scattering events from large domains do not interfere. And therefore, the procedure of incoherent averaging over all domains is just reduced to the substitution of cosγ and $\sin^{2}\gamma$ by their mean values ${\langle \cos\gamma \rangle }_{\mathrm{inc}}$ and ${\langle \sin^{2}\gamma \rangle }_{\mathrm{inc}}$, respectively, averaged over angles γ between magnetization directions in domains and external field. The subscript inc indicates that the averaging is performed incoherently. This averaging can easily be accomplished because the absolute values of magnetization in each of the domains is, by definition, the same and equal to that of the saturation magnetization.

Taking into account that ${\langle \sin^{2}\gamma \rangle }_{\mathrm{inc}}=1-{\langle \cos^{2}\gamma \rangle }_{\mathrm{inc}}$ one can also introduce the dispersion:

(6) $$\Delta =\sqrt{{\langle \cos^{2}\gamma \rangle }_{\mathrm{inc}}-{\langle \cos\gamma \rangle }_{\mathrm{inc}}^{2}},$$

which, together with the expectation value ${\langle \cos\gamma \rangle }_{\mathrm{inc}}$ quantifies basic statistical properties of magnetization distribution over an ensemble of domains. Indeed, in a single domain state, Δ is always zero at any magnetization orientation determined by the parameter ${\langle \cos\gamma \rangle }_{\mathrm{inc}}$. If, alternatively, the sample is totally demagnetized via a set of $180^{\circ }$ domains so that ${\langle \cos\gamma \rangle }_{\mathrm{inc}}=0$, then $\Delta ={\langle \cos^{2}\gamma \rangle }_{\mathrm{inc}}^{1/2}\neq 0$. In general a multidomain state is characterized by 0≤Δ≤1 and $-1\leq {\langle \cos\gamma \rangle }_{\mathrm{inc}}\leq 1$. For domain magnetizations equally distributed over all in-plane directions, the dispersion Δ=1/2, while ${\langle \cos\gamma \rangle }_{\mathrm{inc}}=0$. Note that both ${\langle \cos^{2}\gamma \rangle }_{\mathrm{inc}}$ and ${\langle \cos\gamma \rangle }_{\mathrm{inc}}^{2}$ are observables that can be gained from fitting the PNR data. Hence, the dispersion Δ yielding information on the domain state of the sample is a most instructive quantity to be easily extracted from PNR measurements.

**Figure 2:**
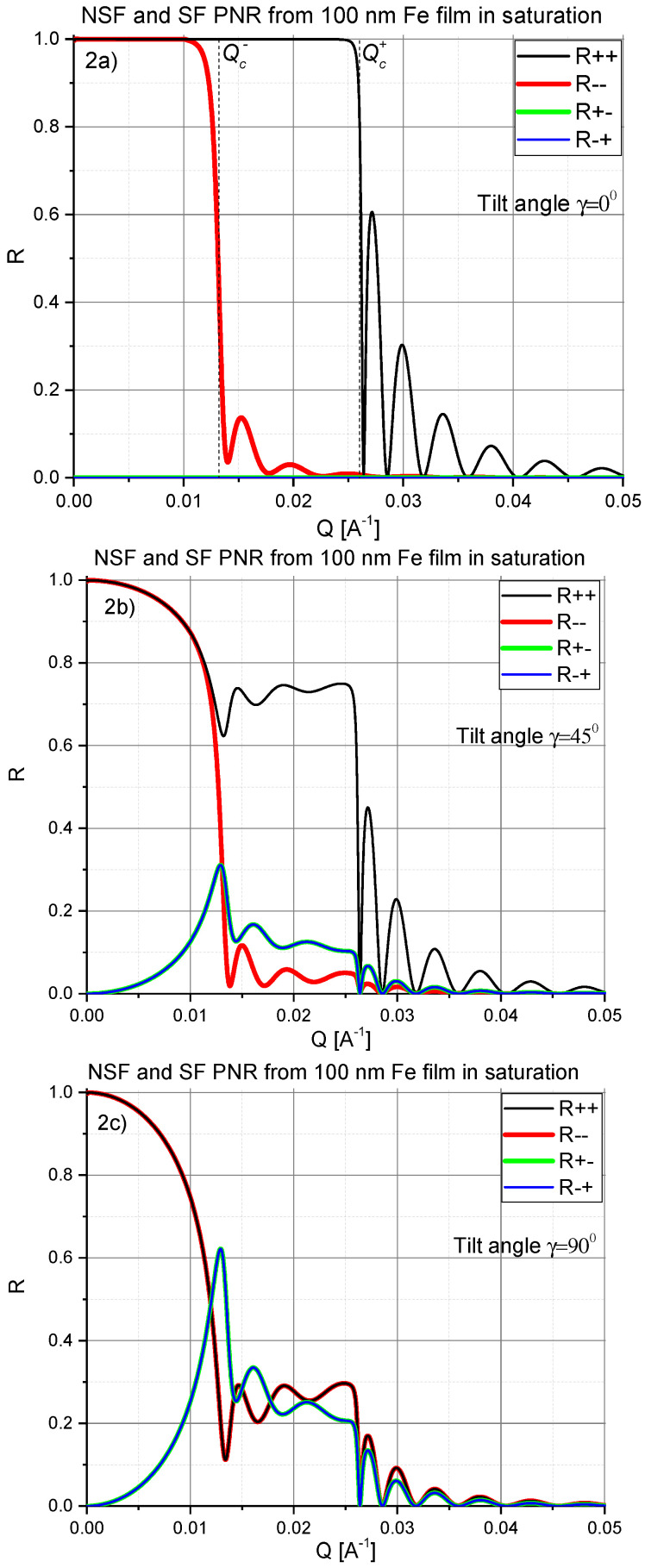
NSF, $R^{++}$ (black solid line) and $R^{--}$ (red solid line), and SF, $R^{+-}=R^{-+}$ (blue lines), reflectivities calculated for a 100 nm homogeneously magnetized iron film on a silicon substrate are shown for the magnetization vector oriented: (**a**) parallel to the axis *Y*, (**b**) tilted by the angle $\gamma =45^{\circ }$ and (**c**) for $\gamma =90^{\circ }$. The positions of the critical reflection edges $Q_{c}^{\pm }$ (independent of γ) are manifested by sharp drops of both NSF reflectivity curves. The position $Q_{c}^{-}$ in (**b**,**c**) is confirmed by the sharp maximum in SF reflectivities. Here *Q* is the modulus of the scattering vector defined by: Q=(4π/λ)sinα.

**Figure 3:**
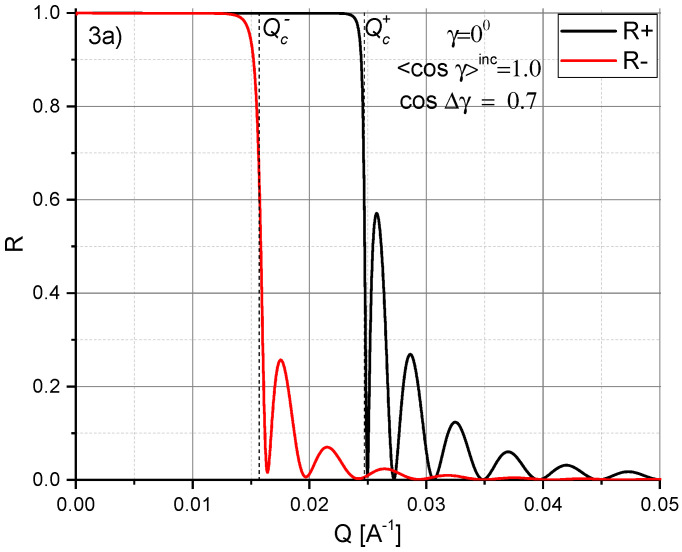

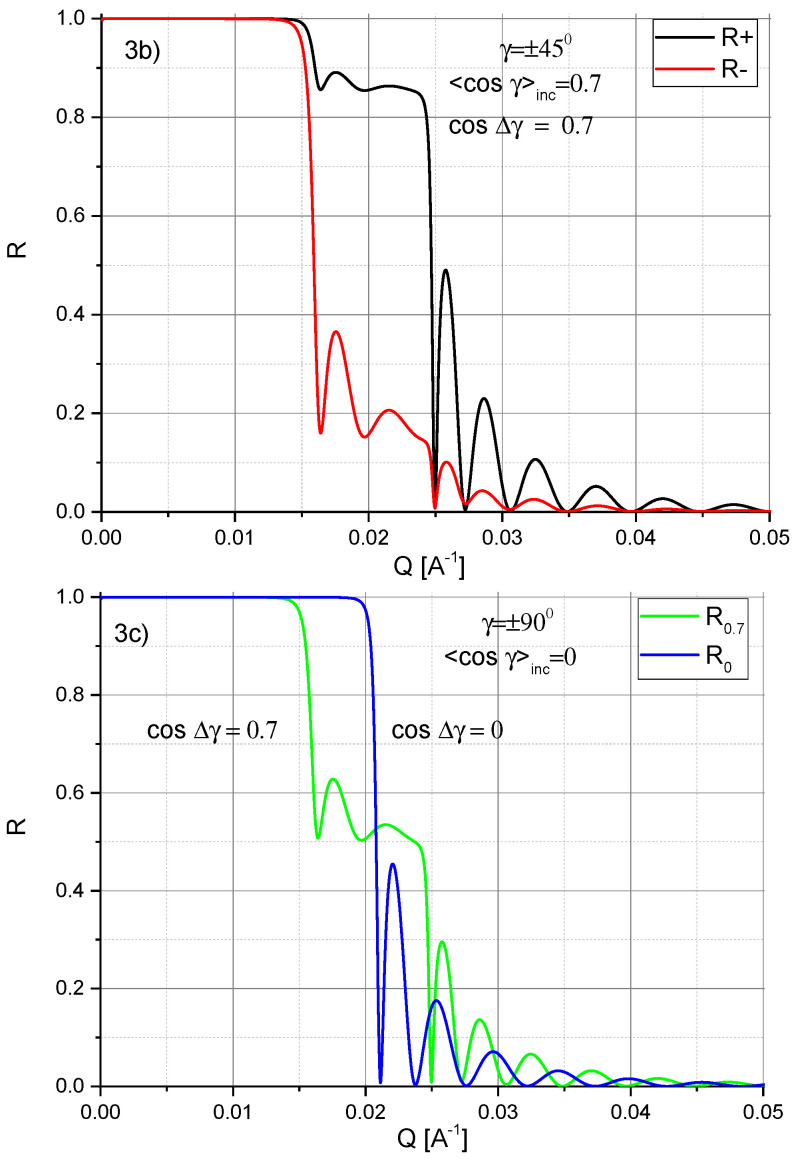
Calculated specular reflectivities $R^{+}$ and $R^{-}$ for a 100 nm thick iron film with reduced magnetization. (**a**) $R^{+}$ (black solid line) and $R^{-}$ (red solid line) for a mean magnetization $\bar{M}=0.5M^{\mathrm{sat}}$ parallel to the Y-axis ($\gamma =0^{\circ }$); (**b**) similar to (**a**) but with the mean magnetization tilted by the angle $\gamma =\left(\pm \right)45^{\circ }$; (**c**) green line: similar to (**a**) but with the mean magnetization tilted by the angle $\gamma =\left(\pm \right)90^{\circ }$; blue line: reflectivity for a mean magnetization $\bar{M}=0$.

It is also important to note that the averaging procedure over large domains does not affect the reflection amplitudes $r_{\pm }$, which according to Equations (1)–(5) are determined by the SLDs $Nb_{\pm }=Nb_{n}\pm Nb_{m}$. Here the magnetic SLD $Nb_{m}$ is proportional to the saturation magnetization, in accordance with Equation (3). Therefore, the location of the critical edges of total reflection

(7) $$Q_{c}^{\pm }=4\sqrt{\pi (Nb_{n}\pm Nb_{m})},$$

as well as the reflection amplitudes are *independent* of the magnetization direction, as is illustrated in Figure 2. The locations of the critical edges $Q_{c}^{\pm }$ are marked in the top panels of Figure 2 and Figure 3.

To exemplify our discussion so far, in Figure 2, a set of reflectivity curves $R^{++}$, $R^{--}$ and $R^{+-}=R^{-+}$ are calculated for a 100 nm thick iron film deposited onto a silicon substrate and for magnetization vector orientations: (a) parallel to the axis *Y*, (b) tilted to the angle $\gamma =45^{\circ }$ and (c) to the angle $\gamma =90^{\circ }$ with respect to the *Y* axis. All reflectivities are shown on a linear scale for better visibility of the behavior close to the critical edges, instead of the more common logarithmic scale.

In Figure 2a one can readily recognize plateaus of totally reflected intensity for each of NSF reflectivity curves, so that $R^{--}$ steeply decreases at $Q_{z}\geq Q_{c}^{-}$, while $R^{++}$ abruptly drops down when $Q_{z}\geq Q_{c}^{+}$, hence indicating positions of corresponding critical reflection edges $Q_{c}^{\pm }$. The width of the plateau, or more precisely the difference ${\left(Q_{c}^{+}\right)}^{2}-{\left(Q_{c}^{-}\right)}^{2}$ is proportional to the saturation magnetization $M_{sat}$, according to Equations (3) and (7).

Figure 2b,c show that total NSF reflection (R = 1) does not exist at $Q_{z}\neq 0$ if the angle between the magnetization direction and the *Y*-axis γ≠0. However, the positions of the critical edges $Q_{c}^{\pm }$, determined by steep descent in reflectivity curves, are *totally independent* of the angle γ, although the intensity distribution between different NSF and SF reflection channels substantially changes with an angle γ. Note, that the position $Q_{c}^{-}$ in Figure 2b,c is also confirmed by the sharp maximum in SF reflectivities.

If, on the other hand, the magnetic domain sizes turn out to be much smaller than the coherence volume and the latter comprises a number of domains, then two prominent effects emerge. The first and the most distinct one is off-specular scattering (OSS) [17], which allows to measure e.g., the domain size and will be discussed later. The second effect is a reduction of the mSLD due to the destructive interference of neutron waves scattered from different domains in directions of specular reflection and transmission. Such a reduction is accounted for via replacing the mSLD in Equation (3) by its mean value. This averaging within a coherence volume is indicated by a overline bar. The equation ${\bar{Nb}}_{m}=C\bar{M}$ then links the mean SLD ${\bar{Nb}}_{m}$ and mean magnetization $\bar{M}$ averaged over the coherence volume. Such averaging can be easily performed in case that the domain magnetization is tilted at angles Δγ to the left or to the right with respect to the direction of the mean magnetization vector $\bar{M}$ of the coherence volume. Then the cartesian projections of this vector are: ${\bar{M}}_{y}=M^{\mathrm{sat}}\bar{\cos\Delta \gamma }$ and ${\bar{M}}_{x}=M^{\mathrm{sat}}\bar{\sin\Delta \gamma }$. Taking into account that the direction of the vector $\bar{M}$ is fixed by the condition $\bar{\sin\Delta \gamma }=0$, one can conclude that $\bar{M}=M^{\mathrm{sat}}\bar{\cos\Delta \gamma }\leq M^{\mathrm{sat}}$.

For domains smaller than the coherence volume, the reduction of the magnetic SLD also affects the positions of the critical edges $Q_{c}^{\pm }$ for total reflections, in contrast to the case of large domains discussed before. Therefore, in Equation (7) the mSLD $Nb_{m}$ has to be substituted for its mean value ${\bar{Nb}}_{m}$. Consequently, the NSF and SF reflectivities, determined by their mean reflection amplitudes ${\bar{r}}_{\pm }$, depend on the mean mSLD ${\bar{Nb}}_{m}$ which, in turn, may also depend on the applied field.

In neutron reflectometry the coherence area, which is the intersectional area of the coherence volume with the sample surface, usually covers only a very small fraction of the sample surface illuminated by the neutron beam, as illustrated in Figure 1. Therefore, the measured reflection is the result of incoherent averaging over a huge number of coherence ellipsoids. This additional averaging is trivial, as long as the mean magnetization vector $\bar{M}$ is the same for each coherence ellipsoid and directed along with external field parallel to the polarization axis. Then, instead of Equations (1) and (2), one has:

(8) $$R^{++}=|{\bar{r}}_{+}{|}^{2}/2,\;\;\;R^{--}={\left|{\bar{r}}_{-}\right|}^{2}/2\;\;\;\mathrm{and}\;\;\;R^{\pm \mp }=0.$$

If the vector $\bar{M}=\left|\bar{M}\right|\cos\gamma$, being identical for each of coherence volumes, is not, however, parallel to *Y*-axis, but tilted by the angle γ with respect to the neutron polarization direction, then NSF and SF reflectivities are determined by the same set of Equations (1) and (2) with reflection amplitudes $r_{\pm }$ replaced by their mean values ${\bar{r}}_{\pm }$.

When, alternatively, the angle γ is not identical for each coherence ellipsoid varying over distances greater than the coherence length, then Equations (1) and (2) NSF and SF reflectivities should be additionally averaged over the angle γ incoherently. The result of such averaging of NSF reflectivities can be represented by the sums of two terms:

(9) $$R^{++}=R^{+}-R^{+-}\;\;\;\;\mathrm{and}\;\;\;\;\;R^{--}=R^{-}-R^{-+},$$

in which the first terms,

(10) $$R^{\pm }=\frac{|{\bar{r}}_{+}{|}^{2}+{\left|{\bar{r}}_{-}\right|}^{2}}{2}\pm \frac{|{\bar{r}}_{+}{|}^{2}-{\left|{\bar{r}}_{-}\right|}^{2}}{2}{\langle \cos\gamma \rangle }_{\mathrm{inc}},$$

describe reflection of polarized neutrons without analysis of final spin states, while the second terms,

(11) $$R^{+-}=R^{-+}=\frac{|{\bar{r}}_{+}-{\bar{r}}_{-}{|}^{2}}{4}{\langle \sin^{2}\gamma \rangle }_{\mathrm{inc}},$$

correspond to the SF reflection coefficients.

The PNR mode discussed so far is referred to as full one-dimensional (1D) analysis of the neutron polarization, which yields the most complete information for flat samples with in-plane magnetization. In the reduced mode of PNR, the incident neutron beam can be polarized but polarization analysis of the reflected beam is not available. Then the reflected beam is a sum of both contributions NSF and SF reflectivity and therefore only two reflectivities, $R^{+}$ and $R^{-}$, can be determined and described by Equation (10), while any information related with SF reflection described by Equation (11) is missing.

For the trivial case of a homogeneously magnetized sample in a single domain state, both modes of PNR are equivalent and yield the same information as both contain the same set of parameters. In either case, the main information to be extracted is the SLDs $Nb_{n}$, $Nb_{m}$ and the magnetization tilt angle γ. However, a homogeneous sample magnetization is rarely a subject of interest for PNR studies, as saturation magnetization and its tilt angle can and should be measured with easier available laboratory magnetometers. Vibrating sample magnetometer (VSM) and SQUID measures the net magnetization projection onto the field direction and are not sensitive to its distribution over the sample surface. On the other hand, PNR carries additional valuable information on domain dimensions and domain dispersion scaled with the neutron coherence range.

Even in the reduced mode of PNR, one may clearly discriminate between a magnetization that is averaged over small domains and a magnetization averaged over dimensions larger than the coherence length. In the first case, the positions of the critical edges for total reflection are affected, while in the second case the critical edges are unaffected while the intensities in the plateau region are different for both spin components.

For illustrating the reduced reflectivity mode, we have calculated reflectivity curves in Figure 3 for the same 100 nm thick iron film as shown in Figure 2 but without polarization analysis of the reflected neutrons, and for different magnetization conditions, as explained in the following. In Figure 3a we assume that the iron film is partially demagnetized, so that the mean magnetization parallel to the Y-axis ($\gamma =0^{\circ }$, ${\langle \cos\gamma \rangle }_{\mathrm{inc}}$ = 1) is $\bar{M}=0.5M^{\mathrm{sat}}$. The respective reflectivities $R^{+}$ (black solid line) and $R^{-}$ (red solid line) are similar to those shown in Figure 2a. However, the difference ${\left(Q_{c}^{+}\right)}^{2}$-${\left(Q_{c}^{-}\right)}^{2}$ is reduced because of the decreased mean magnetization $\bar{M}$.

In Figure 3b is similar to Figure 3a but with the mean magnetization $\bar{M}$ tilted by the angle $\gamma =\left(\pm \right)45^{\circ }$. Again, the difference between $Q_{c}^{+}$ and $Q_{c}^{-}$ is reduced in comparison to the one in Figure 2b. Furthermore, the sharp maximum seen in Figure 2b at $Q_{c}^{-}$ is now incorporated in the reflectivity $R^{-}$.

Figure 3c illustrates two cases. The green line shows the reflectivity for a homogeneous magnetization $\bar{M}=0.5M^{\mathrm{sat}}$, tilted by $\gamma =\left(\pm \right)90^{\circ }$. The corresponding reflectivities $R^{+}=R^{-}$ are merged, but the steep drops of reflected intensity at $Q_{c}^{-}$ and a further drop at $Q_{c}^{+}$ are clear indications for a mean magnetization that is tilted by $\gamma =\left(\pm \right)90^{\circ }$. The blue line shows the reflectivity assuming total demagnetization averaged over small domains. Therefore, a tilt of a finite magnetization can clearly be distinguished from a complete demagnetization even without polarization analysis.

**Figure 4:**
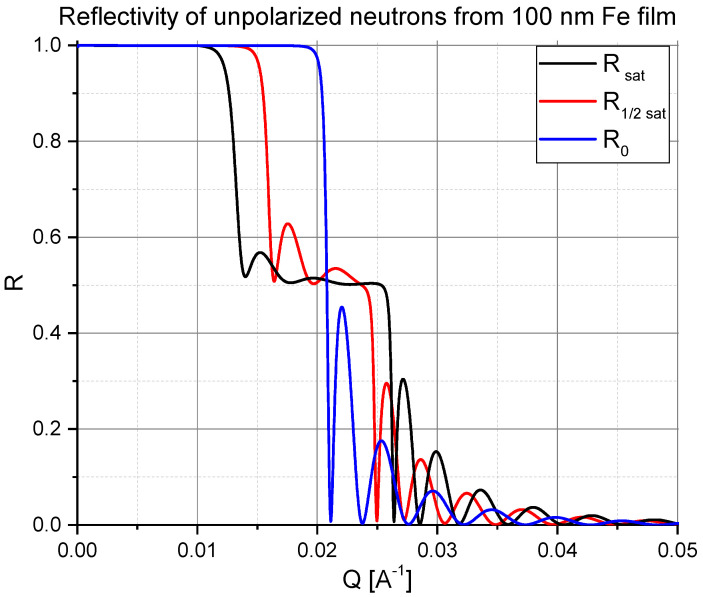
Illustration of unpolarized neutron reflectivities calculated for iron film in saturation (black), on half way to saturation (red) and in totally demagnetized state (blue) via formation of a random set of small domains.

Even *unpolarized* neutron reflectometry is able to measure the mean magnetization averaged over small domains delivering information that is hardly accessible with bulk magnetometry, and different of that provided either by magneto-optic Kerr effect (MOKE), or Lorentz microscopy and magnetic force microscopy (MFM). Indeed, for an unpolarized neutron beam the reflection coefficient $R=(R^{+}+R^{-})/2$ is described by the mean reflectivity:

(12) $$R=\frac{1}{2}\left\{|{\bar{r}}_{+}{|}^{2}+{\left|{\bar{r}}_{-}\right|}^{2}\right\},$$

averaged over neutron spins states. The results of the spin averaging is illustrated in Figure 4 where reflectivity curves are plotted for three values of the mean magnetization: $\bar{M}=M^{\mathrm{sat}}$, $\bar{M}=M^{\mathrm{sat}}/2$ and $\bar{M}=0$. For this simulation, the orientation of the mean magnetization does not matter and cannot be resolved. But the steep drop of the reflected intensity to a plateau region and a second drop before reaching the Kiessig fringes unambiguously indicates that the sample exhibits a finite magnetization. Moreover, the finite magnetization is proportional to the width of the plateau region.

As already mentioned, using unpolarized neutron reflectivity we cannot determine the magnetization tilt angle γ. Strictly speaking, the tilt angle can not be uniquely determined with the reduced mode of PNR since it is sensitive to only the projection of magnetization onto the *Y*-axis characterized by the parameter ${\langle \cos\gamma \rangle }_{\mathrm{inc}}$, but information on the X-projections is missing. Then one cannot distinguish between random distribution of domain magnetization around some single direction from bi-modal distribution characterized by two alternative magnetization directions. This is only possible with full 1D PNR. Therefore aiming for a full polarization analysis is preferable, provided the experimental conditions allow to do so.

It is worth mentioning that PNR is usually considered as a unique method to be applied to record magnetization depth profiles in e.g., multilayers. This is of particular interest in case of a complex noncollinear depth distribution of a 3D magnetization vector. Despite of appreciable progress in this direction, currently, only 1D polarization analysis is experimentally established, but obviously not sufficient to reliably recover depth profiles of all three components of the magnetization vector. Here we do not discuss advantages of the vector (3D, spherical, etc.) polarization analysis techniques and just mention that theoretical tools for description of such experiments are available [18].

For a full treatment of the theory of neutron reflectometry we refer to the reviews listed in Refs. [7,16,19]. In short, PNR from thin magnetic films in saturation parallel to the *Y*-direction shows three salient features, as illustrated in Figure 2:

- A flat intensity plateau due to total reflection for wave-vectors $Q_{z}<Q_{c}^{\pm }$, where $Q_{z}=\left(4\pi /\lambda \right)\sin\alpha_{i,f}$ is the modulus of the scattering vector, $\alpha_{i,f}$ are the glancing angles with $\alpha_{i}=\alpha_{f}$ for specular reflection, and $Q_{c}^{\pm }$ are the critical wave numbers for total reflection defined by Equation (7).
- If the sample is homogeneously magnetized or decomposed into a set of large magnetic domains then the critical edges, which separate the total reflection region from the rest of reflectivity, are distinctly different for up and down polarized neutrons. The difference ${\left(Q_{c}^{+}\right)}^{2}-{\left(Q_{c}^{-}\right)}^{2}$ being proportional to the saturation magnetization $M^{\mathrm{sat}}$ is independent of the angle γ between magnetization direction and *Y*-axis. In contrast, the angle γ determines the reflected intensity distribution between different NSF and SF reflection channels.
- In case of domains smaller than the coherence area the difference between ${\left(Q_{c}^{+}\right)}^{2}$ and ${\left(Q_{c}^{-}\right)}^{2}$ is proportional to the mean magnetization $\bar{M}$ averaged over small domains.
- For $Q_{z}>Q_{c}$, oscillatory Kiessig fringes occur, the period of which at $Q_{z}\gg Q_{c}$ is inversely proportional to the film thickness, while the reflected intensity drops according to $∼Q_{z}^{-4}$.

In what follows, we will focus on magnetic thin films broken down into periodic stripe arrays in the lateral direction. But before going into further details, the concept of the coherence volume for neutron reflectivity and its application to stripe arrays will be emphasized, which is essential for interpreting experimental results of patterned samples.

### 2.2. Coherence Volume

Most modern neutron reflectometers, steady state or time-of-flight, are equipped with a rectangular narrow entrance slit for the incident beam and with a position sensitive detector (PSD) measuring the exit beams. Assuming that the scattering plane is the horizontal *Z*-*X* plane, the short side of the entrance slit is oriented along the *Z*-direction as seen in Figure 1, defining the glancing angles $\alpha_{i}$ of incidence. The PSD covers the *Z*-*Y* plane and the pixel position on the PSD defines the exit (final) angle $\alpha_{f}$ between scattered beam and the surface. The long and wide open entrance slit parallel to the *Y*-direction enhances the intensity, which is often additionally enhanced by focusing the incident beam onto the sample surface. By geometry, the angular resolutions (uncertainties) are drastically different for both directions: $\Delta \theta_{x}$ = $\Delta \alpha_{i}$ + $\Delta \alpha_{f}\ll \Delta \theta_{y}$. The coherence ellipsoid is the volume in real space within which scattering *amplitudes* are added up and constructive/destructive inference takes places. Beyond the coherence volume, reflected *intensities* from different coherence volumes are added up to yield the total reflected intensity from a sample surface. The coherence ellipsoid is defined by the uncertainties $\Delta \alpha_{i}$, $\Delta \alpha_{f}$, $\Delta \theta_{y}$, and by the wavelength λ. Because of the collimation conditions, as already mentioned, the coherence volume is highly anisotropic, but its anisotropy dramatically increases due to shallow angles of incidence, $\alpha_{i}$, and scattering, $\alpha_{f}$. Moreover, very strong anisotropy occurs even in case of a pin hole collimation usually employed in Grazing Incidence Small Angle Neutron Scattering (GISANS) experiments [20].

Coherence properties are usually quantified by the coherence ellipsoid reciprocal to the resolution ellipsoid with principal axes $\Delta Q_{x}$, $\Delta Q_{y}$ and $\Delta Q_{z}$. Hence, the longest axis of the coherence ellipsoid, or coherence length $l_{x}^{\mathrm{coh}}∼2\pi /\Delta Q_{x}$, is determined by the uncertainty $\Delta Q_{x}$ in the lateral projection $Q_{x}$ of the wave vector transfer *Q* and can be estimated as:

(13) $$l_{x}^{\mathrm{coh}}∼\frac{\lambda }{\sqrt{{\left(\alpha_{i}\Delta \alpha_{i}\right)}^{2}+{\left(\alpha_{f}\Delta \alpha_{f}\right)}^{2}}}.$$

Under experimental conditions, the coherence length $l_{x}^{\mathrm{coh}}$ ranges typically from a fraction of a millimeter down to a few micrometers.

The shortest axis of the coherence ellipsoid is determined by the uncertainty $\Delta Q_{y}$ in the wave vector transfer component $Q_{y}$ and this is much higher than $\Delta Q_{x}$. Thus, the shortest axis is estimated as

(14) $$l_{y}^{\mathrm{coh}}∼\lambda /\Delta \theta_{y}\ll l_{x}^{\mathrm{coh}}$$

and the coherence length in this direction does not exceed 10 nm.

**Figure 5:**
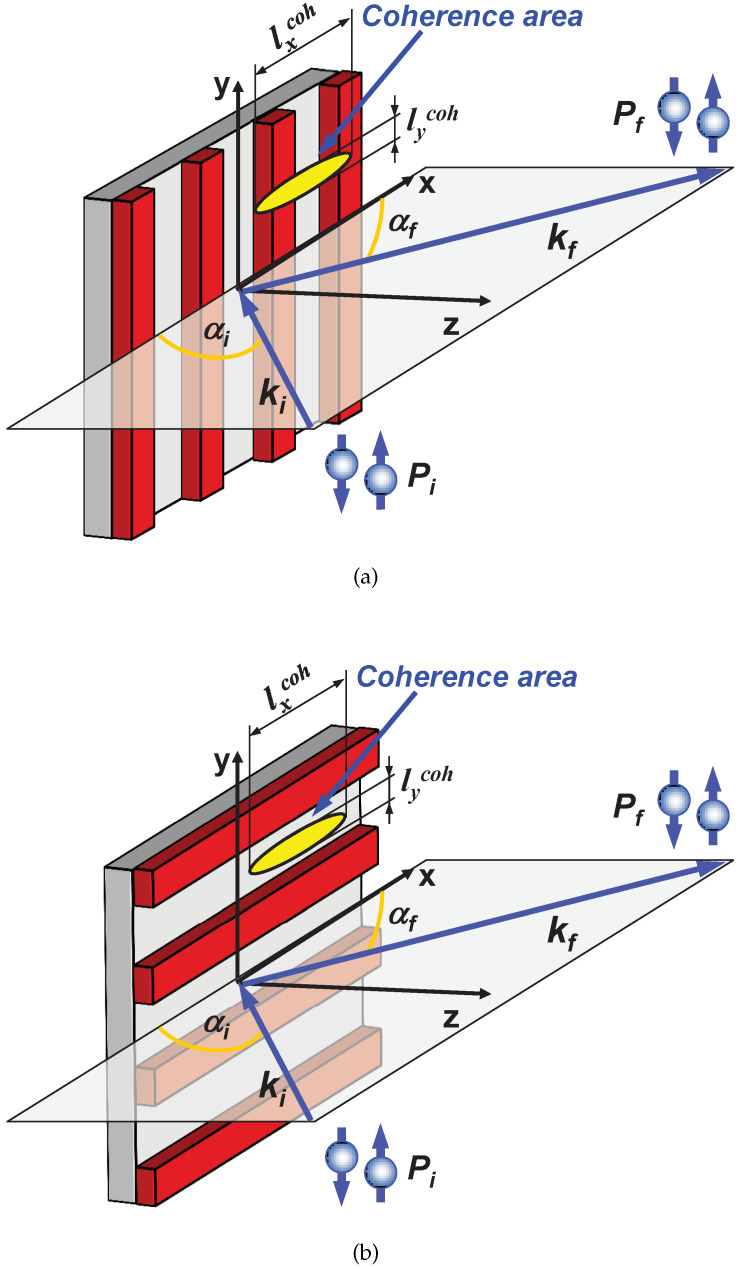
Mutual orientations of the coherence ellipsoid and the array of micro-stripes. (**a**) The coherence area crosses several stripes and off-specular Bragg diffraction can be observed. (**b**) The coherence area is collinear with the stripes, resulting solely in specular reflection, assuming flat edges and flat surfaces of the stripes.

The length of the third axis is determined by the uncertainty $\Delta Q_{z}$ in the wave vector transfer component $Q_{z}$ normal to the surface and can be estimated by the uncertainties of the incident and exit angles:

(15) $$l_{z}^{\mathrm{coh}}∼\lambda /\sqrt{{\left(\Delta \alpha_{i}\right)}^{2}+{\left(\Delta \alpha_{f}\right)}^{2}},$$

so that the coherence length $l_{z}$ is estimated to be in the order of a fraction of micrometer, and hence $l_{y}^{\mathrm{coh}}\ll l_{z}^{\mathrm{coh}}\ll l_{x}^{\mathrm{coh}}$. The coherence ellipsoid is indicated schematically in Figure 5 on a thin film patterned with a periodic lateral micro-stripe array.

### 2.3. Stripe Array with Parallel and Perpendicular Orientation

For orientation of the micro-stripes perpendicular to the scattering plane, as depicted in Figure 5a, the large extension of the coherence volume $l_{x}^{\mathrm{coh}}$ covers a number of stripes. This is a prerequisite for coherent enhancement of scattering amplitudes whenever the Bragg equation $Q_{x}=2\pi n/d$ is satisfied, where the integer *n* numerates the order of Bragg reflection in the lateral direction, and *d* is the stripe array period of a few micrometers.

At a given shallow incident angle $\alpha_{i}>0$, Bragg scattering is excited when the shallow scattering angle $\alpha_{f}=\alpha_{-}^{B}>0$, where

(16) $$\alpha_{-}^{B}\approx \sqrt{\alpha_{i}^{2}-2n\lambda /d}>\alpha_{i},$$

with integer n<0. Alternatively, Bragg conditions are satisfied when $\alpha_{i}=\alpha_{+}^{B}$, where

(17) $$\alpha_{+}^{B}\approx \sqrt{\alpha_{f}^{2}+2n\lambda /d}>\alpha_{f},$$

with n>0. For n=0 these equations merge with Snell’s law $\alpha_{f}=\alpha_{i}$ for specular reflection, the amplitude of which is determined by the mean SLD averaged over the coherence ellipsoid. The Bragg reflections and the diffuse scattering observed at $\alpha_{f}\neq \alpha_{i}$ is referred to as off-specular scattering (OSS). The Bragg reflections are generated by periodic deviations $\Delta Nb=Nb-\bar{Nb}$ of SLD from its mean value $\bar{Nb}$, while the diffuse scattering occurs due to random SLD deviations.

Now we consider the case that an external field *H* applied parallel to the *Y*-axis is high enough to saturate the magnetization within the stripes for the parallel and the perpendicular orientation. As the field *H* is homogeneous along the *Y*-axis, it does not cause scattering contrast. Therefore we conclude that for perpendicular orientation of the stripes, the mean SLD value is given by ${\bar{Nb}}^{\pm }=\eta Nb^{\pm }$, where η=w/d is the surface fraction covered by the stripes. These reduced mean SLDs are used to calculate the reflection *amplitudes* ${\bar{r}}^{\pm }$ for each of the spin states. Then NSF and SF reflection coefficients and corresponding OSS cross sections are averaged over incident and outgoing spin states filtered by the polarizer and analyzer. The result is convoluted with the 3D resolution function and incoherently averaged over all coherence ellipsoids spanning the whole sample surface.

Thus, in perpendicular orientation, specular reflection and Bragg diffraction at shallow angles provides information on width and periodicity of stripe arrays and on magnetic induction within the stripes. In this orientation OSS is, however, less sensitive to the possible inhomogeneity of magnetic flux in stripes with not ideal edges and, especially, in the *inter*-space [21]. Therefore, a complimentary parallel orientation has to be chosen to supplement the remaining information.

In parallel orientation, the stripe pattern is rotated by 90${}^{\circ }$ about the surface normal and the long axis of the coherence ellipsoid lies now parallel to the stripes. In this orientation sketched in Figure 5b, no Bragg diffraction can be seen as neither of coherence ellipsoids crosses more than a single stripe. Then neutron waves scattered from different stripes do not interfere. Also there is almost no interference between waves reflected from the stripes and from the inter-stripe spaces filled with magnetic field. As a result, the specular reflection coefficients $R^{\pm }$ can be represented as an incoherent sum of reflectivities from individual stripes $R_{\mathrm{stripe}}^{\pm }$ and reflectivities $R_{\mathrm{inter}}^{\pm }$ from the inter-stripe regions weighted with the corresponding surface factors η and (1−η), respectively:

(18) $$R^{\pm }=\eta R_{\mathrm{stripe}}^{\pm }+\left(1-\eta \right)R_{\mathrm{inter}}^{\pm }.$$

This relation holds independent on whether the magnetization of the stripes is in a saturated state parallel or perpendicular to the stripes. But the magnetization orientation will affect the proportion between NSF and SF specular reflectivity and off-specular scattering. If, on the other hand, the magnetization is not constant and split up into domains, in addition off-specular diffuse scattering will be observed that yields information on the domain size and distribution, as already discussed for homogeneous flat magnetic films.

Summarizing, both orientations of stripe arrays, parallel and perpendicular to the scattering plane, are needed for a complete analysis of the stripe patterns. The perpendicular orientation yields information on the geometry of the stripe array, including stripe width and periodicity, whereas the parallel orientation adds information on the magnetic flux distribution in e.g., the *inter*-space region.

## 3. Experimental Study of a Magnetic Stripe Array

**Figure 6:**
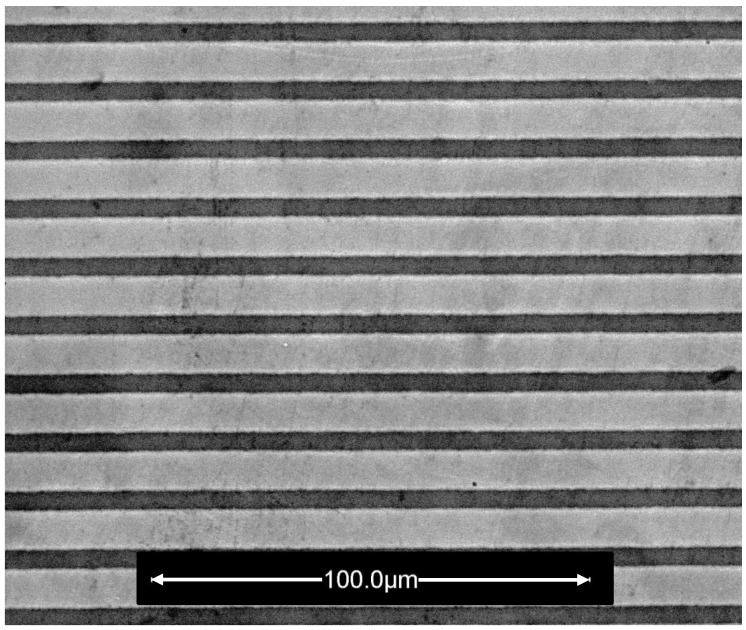
Scanning electron microscopy image of the Py stripe array studied by PNR. The Py stripes appear in light gray, the spaces in between show a dark-gray shade.

In the following we present PNR and OSS measurements performed on a magnetic stripe array. The sample with the size of 8 × 12 mm${}^{2}$ consists of an array of parallel permalloy ($Fe_{20}Ni_{80}$) stripes with a nominal width of w=7.5μm, lateral period of d=13.3μm and nominal permalloy thickness of 800 Å. The sample was capped with a protective Cr layer against oxidation. The stripes were fabricated on a single-crystalline Si(100) substrate and oriented parallel to the longer edge of the sample. The artificial pattern was fabricated by a top-down lithographic process using a Cr photomask containing the pattern design. Figure 6 shows a scanning electron microscopy image of a small representative are of the stripe array. More information on the sample preparation procedures is provided in Ref. [21].

PNR and OSS experiments were performed using the Super-ADAM polarized neutron reflectometer [22] situated at the Institut Laue-Langevin in Grenoble, France. The instrument operates at a constant wavelength λ=4.41 Å with a wavelength spread Δλ/λ=0.7% and an angular divergence of the incident beam $\Delta \alpha_{i}\approx 0.2$ mrad. At each glancing angle of incidence $0<\alpha_{i}<40$ mrad the scattered intensity was recorded via a 2D position sensitive detector (PSD) covering about the same range of glancing scattered angles $\alpha_{f}$ with an uncertainty $\Delta \alpha_{f}\approx 0.3$ mrad in the horizontal (X,Z) scattering plane. In the vertical plane perpendicular to the scattering plane the incident beam was focused at the sample position, providing a divergence $\Delta \theta_{Y}∼50$ mrad in angles $\theta_{Y}$. Polarization analysis was achieved by using a PSD detector combined with a wide-angle multichannel spin analyzer [23].

As introduced above, two complementary sample orientations with respect to the stripes were probed. An external field of 5.2 kOe was applied perpendicular to the scattering plane along the stripes for the perpendicular orientation of the stripes and across the stripes for the parallel orientation with respect to the scattering plane. In both orientations the applied field exceeded the saturation field. In addition, we present also results for the parallel orientation with varying fields from remanence to the saturated state.

**Figure 7:**
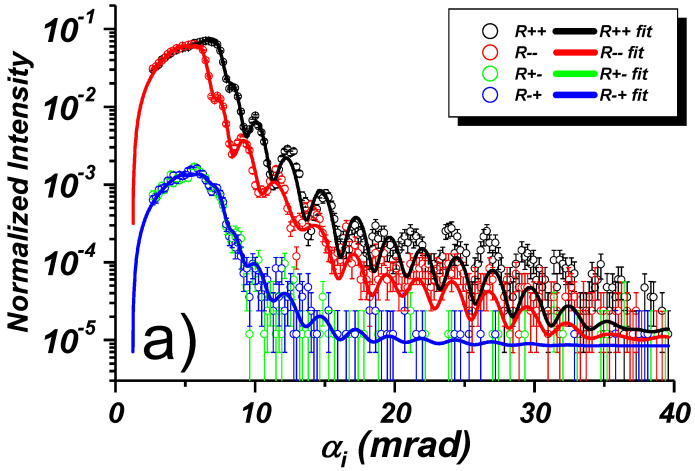

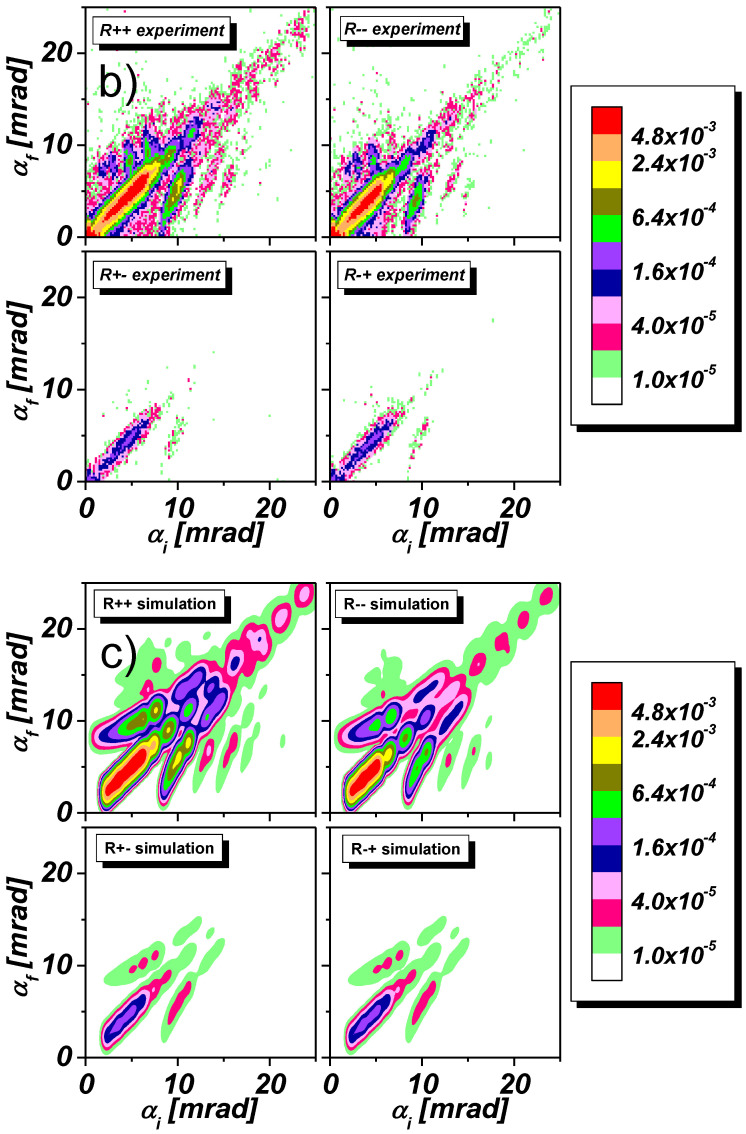
(**a**) NSF and SF specular reflectivities with least square fit from 800 Å thick permalloy stripe array with stripes oriented perpendicular to the scattering plane and in a field of $H_{ext}=5.2$ kOe applied along the stripes. (**b**) Off-specular scattering maps collected simultaneously with the specular reflectivities using a wide-angle multichannel spin analyzer. (**c**) Corresponding model calculations.

Figure 7a shows the results of four specular reflectivity scans $R^{++}$ (black), $R^{--}$ (red), $R^{+-}$ (green), $R^{-+}$ (blue) together with least square fits (solid lines) from the stripe array oriented perpendicular to the scattering plane and in a field of 5.2 kOe applied along the stripes. In Figure 7b off-specular scattering maps are shown, collected simultaneously with the specular reflectivities. In the maps, the glancing incident angle $\alpha_{i}$ is plotted on the x-axis, the exit angle $\alpha_{f}$ on the y-axis, and the intensity is color coded. The specular reflected intensity runs along the diagonal: $\alpha_{i}$ = $\alpha_{f}$. Off-specular scattering (OSS) is seen above and below the specular intensity ridge. Simulations of the maps based on the sample stack model extracted from the fits of the specular reflectivities are displayed in Figure 7c.

The specular NSF reflectivity curves $R^{++}$, $R^{--}$ in panel (a) are clearly split, indicating the magnetic state of the sample. Below the critical angles for total reflection, the plateau region is curved due the diminishing footprint of the incident beam with decreasing $\alpha_{i}$. In the saturated state no SF reflectivity is expected. Indeed, the SF $R^{+-}$, $R^{-+}$ reflectivities are on a 1% level of the NSF reflectivities, and attributed to the non-perfect neutron spin filtering by the polarizer and analyzer.

In the maps shown in panel (b) one can clearly distinguish the off-specular Bragg bands up to third order embracing the specular ridge from both sides as expected due to Equations (16) and (17). Note that the experimentally observed Bragg bands in the upper half-plane characterized by $\alpha_{i}>\alpha_{f}$ are smeared out because of non-centered positioning of the spin analyzer. Due to the large lateral period, the Bragg bands run close to each other and, because of finite resolution of the instrument, merge with the specular reflectivity ridge already at the incident angle of about $\alpha_{i}\approx$ 12 mrad. This merging eventually causes problems when fitting the specular reflectivities shown by the black and red lines in panel (a).

The overlap of OSS and specular reflectivity usually causes a phase shift of the Kiessig fringes. In the present case, the effect is not dramatic and barely seen. In any case, theoretical models for the reflectivity fit cannot describe the phase shifted Kiessig fringes. Reliable results can be obtained only when data in the region $\alpha_{i}≲$12 mrad are used in the fit. The limited fitting range may, however, substantially affect the reliability of the resulting fitting parameters. In particular, when cutting off data in the tail of the reflectivity curve. Then one loses, in particular, information on the Debye-Waller factor describing interface and surface roughness and on low frequency modulations due to the thin cap layer. This missing information can partially be recovered via experiments with the sample rotated by 90${}^{\circ }$ about the normal to its surface, as discussed below.

The simulated maps in Figure 7c well reproduce the main features of the experimental intensity maps. The simulation has been performed within the framework of the Distorted Wave Born Approximation (DWBA) [7,16,19] where neutron wave functions are calculated for mean layer SLDs averaged over coherence ellipsoids, while the periodic structure is considered as a perturbation. Hence the simulations are based on set of parameters deduced from fits of corresponding specular reflectivity curves shown in the Figure 7a. This set includes parameters describing mean SLD structure along *Z*-direction, while further fitting of the intensity distribution over angles $\alpha_{f}\neq \alpha_{i}$ in OSS maps delivered lateral structure parameters, e.g., the stripe width *w* and the array period *d*.

**Figure 8:**
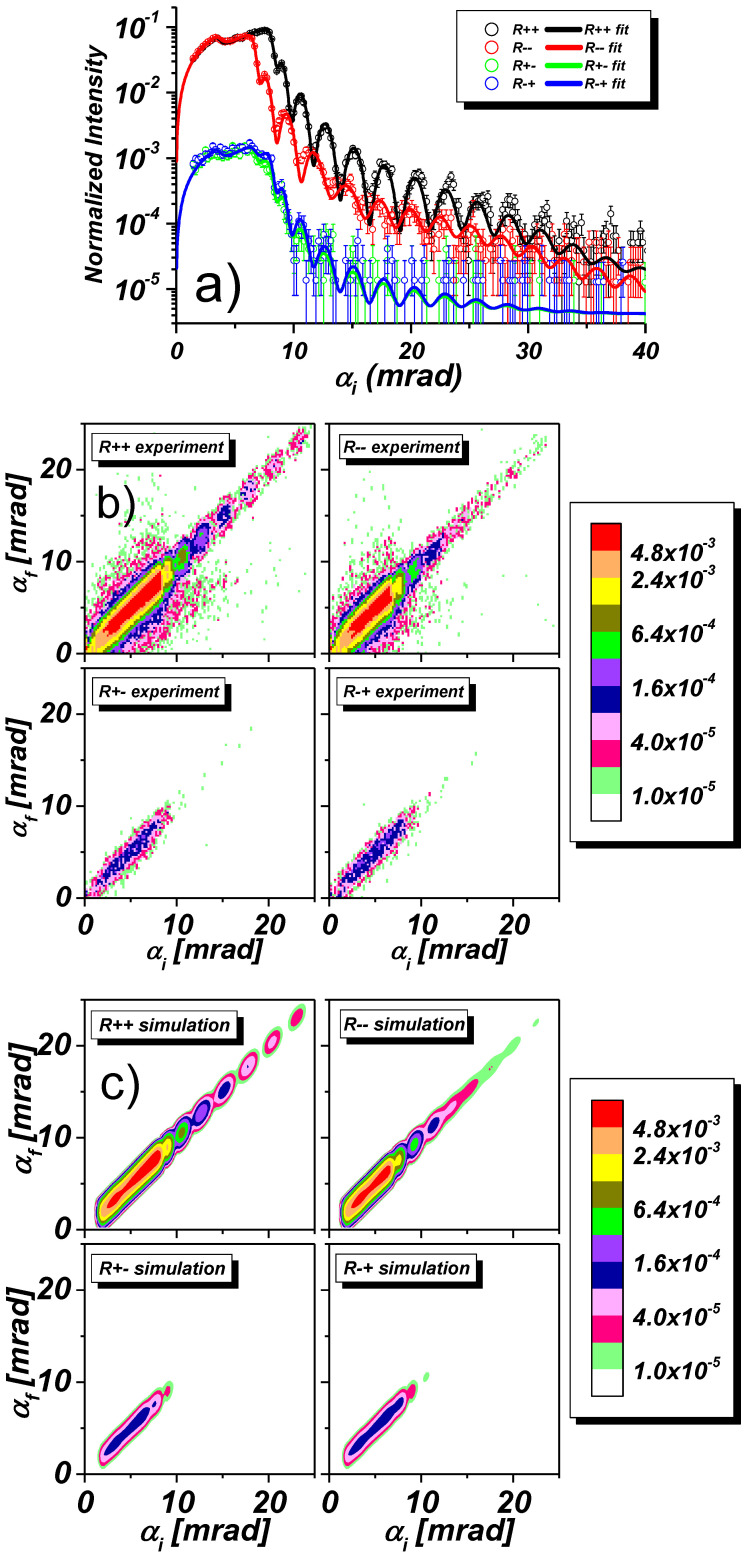
(**a**) NSF and SF reflectivities for the stripes oriented parallel to the scattering plane in a field of $H_{ext}=5.2$ kOe applied across the stripes. (**b**) Off-specular scattering maps and (**c**) corresponding model calculations.

Figure 8 represents fitted specular reflectivities and off-specular scattering maps collected from the sample with stripes oriented parallel to the scattering plane as sketched in Figure 5b. During these measurements, an external field of 5.2 kOe was applied perpendicular to the stripe array. The specular reflectivity scans in panel (a) show again a splitting between the NSF reflectivities $R^{++}$ and $R^{--}$ and negligible SF reflectivities $R^{+-}$ and $R^{-+}$ on the 1% level. In the plateau region below the critical edge for the permalloy, a dip can be seen, which is due to the total reflection from the bare Si substrate, probed by coherence ellipsoids in the inter-stripe space.

The maps in panel (b) show the specular ridge and negligible diffuse scattering, but no trace of Bragg bands. This is expected and accounted for by a coherence ellipsoid precisely oriented parallel to the stripe array with a coherence length $l_{y}$ that covers much less than a stripe width and inter-stripe space. The missing Bragg bands also guarantee a much more reliable fit of the specular reflectivities in panel (a) covering the entire scanned angular range. The low intensity diffuse halos that can be seen in the maps below the critical edge are attributed to small angle scattering from polycrystalline grains of the front aluminium window of the ${}^{3}$He PSD-detector.

According to these maps one can conclude that this sample does not show any detectable off-specular diffuse scattering, which otherwise might originate from fluctuations of the magnetic induction between stripes [21]. This confirms that the rather strong inter-stripe induction $B_{\|}\left(Z,Y\right)$ is independent of the *X* coordinate and quite homogeneously distributed with no appreciable random gradients in the *X* direction along the stripes and the incident beam. The presence of such a homogeneous field immediately follows from the fit of the specular reflectivity to the incoherent sum of reflectivities from stripes and from inter-stripe regions, where magnetic induction between the stripes is determined to be almost equal to that of the saturated stripes. Field gradients in two orthogonal *Z* and *Y* directions cannot cause off-specular scattering in this sample orientation. Hence the absence of off-specular diffuse scattering means that the system is translational invariant with respect to its shift along the stripes (*X*-axis) over a distance smaller than, or comparable with the long axis of the coherence ellipsoid. This is possible if edge roughnesses of stripes and, hence, inter-stripe field inhomogeneities are both absent. Or, alternatively, the edge roughness correlation length in the *X*-direction is larger than the coherence length $l_{x}^{\mathrm{coh}}$. Therefore, the intensity maps in Figure 7c were simulated for the nominal lateral structure with smooth stripe edges and, consequently, homogeneous inter-stripe magnetic induction. The simulated maps well reproduce the experimental maps, aside from the halos that are identified as artifacts.

**Figure 9:**
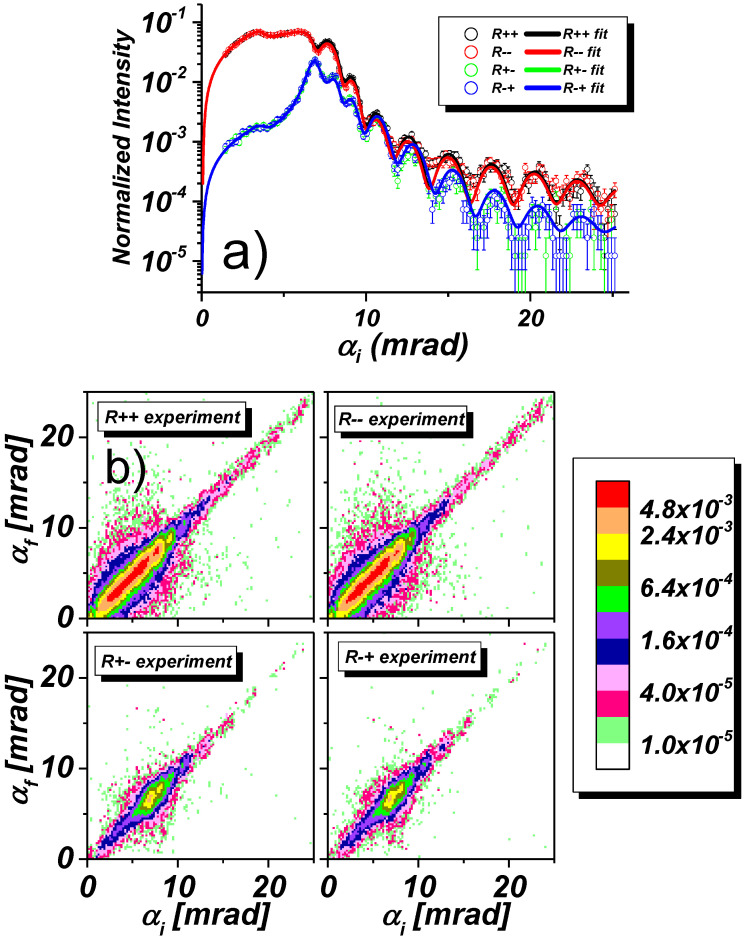
(**a**) NSF and SF reflectivities for the stripes oriented parallel to the scattering plane as in Figure 8 but in a guiding field of $H_{ext}=5$ Oe after the sample was saturated in 5 kOe applied parallel to the stripes. Solid lines are fits to the experimental data. (**b**) Off-specular scattering maps.

So far we have discussed two orientations of the stripe array in a saturation magnetic field of 5.2 kOe parallel to the *Y*-direction and stripes either parallel, or perpendicular to the same axis. The orientation of the stripes affects the presence/absence of Bragg-bands, while SF scattering is absent in both cases. For the occurrence of SF scattering, a magnetic induction component parallel to the *X*-axis is required. But the application of an external magnetic field along the *X*-direction is not possible, as this would depolarize the neutron beam, the initial polarization of which is maintained by a guiding field of minimum 5 Oe applied along the *Y*-axis. One way out is the use of the high aspect ratio of the long stripes combined with the large domain size in Py films. Once magnetized in a vertical saturating field parallel to the the stripe axis, the magnetization is maintained even when this field is reduced down to small fields sufficient to preserve the neutron polarization, while the sample is then rotated by 90${}^{\circ }$ about its normal. Figure 9 shows respective data collected from the same parallel oriented stripe array as in Figure 8, but in a guiding field of 5 Oe. The specular reflectivity in Figure 9a now shows that there is basically no splitting between $R^{++}$ and $R^{--}$, while the SF reflectivities $R^{+-}$ and $R^{-+}$ have much higher intensity approaching the level of NSF reflectivities. Also the intensity maps in Figure 9b show the dramatic enhancement of the SF specular reflection close to the critical edges for permalloy, but with little diffuse scattering. This indicates that the stripes are in a single domain state. The parasitic signal observed in the NSF maps is again due the spurious small angle scattering from the detector window, as mentioned before.

**Figure 10:**
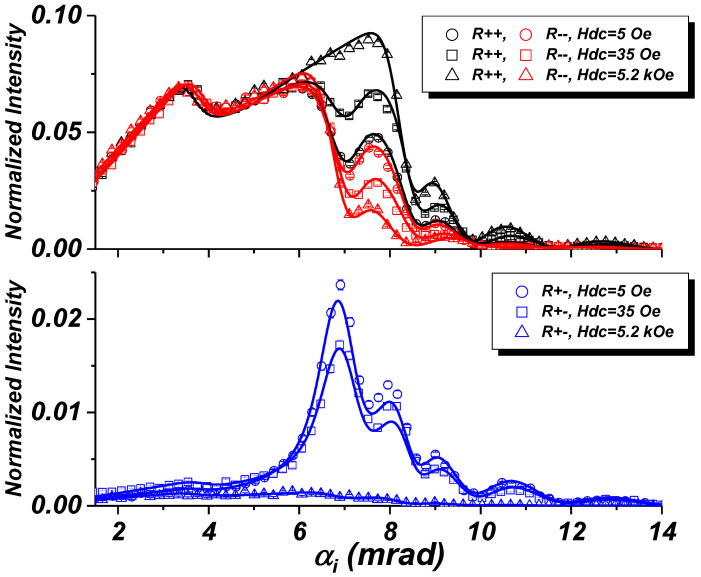
NSF and SF reflectivities plotted on a linear scale for the stripe array oriented parallel to the scattering plane and in different fields applied perpendicular to stripes. Symbols refer to experimental data; solid lines are best fits to the experimental data.

Figure 10 shows a comparison of NSF and SF reflectivities for the parallel orientation of the stripes in different fields applied perpendicular to the scattering plane. The data are shown on a linear scale for easier comparison with Figure 2, but without correction of the finite size of the sample. As a result, the uncorrected NSF experimental curves show a gradual increase of the reflected intensity with increasing incident angle below the total reflection edge, whereas the reflectivities presented in Figure 2 were calculated assuming an infinite sample area resulting in $R^{++}=R^{--}$ = 1 at $\alpha_{i}$ = 0. Note that the intensity scales in panel (a) and (b) are different. The SF reflectivity is highest for the stripe array in the remanent state with the magnetization parallel to the stripes. The SF reflectivity decreases with increasing field in the direction perpendicular to the stripe array and vanishes in the saturating field of 5.2 kOe. Similar, the NSF reflectivities show essentially no splitting at remanence, while the splitting increases with increasing field. The critical edge clearly observed at 3.5 mrad is due to the bare Si substrate, probed by neutrons in the inter-stripe space.

## 4. Summary and Conclusions

Polarized neutron reflectivity on magnetic thin films is well described and often practiced to extract the nuclear and magnetic scattering length density profile normal to the film plane. This includes information on the magnetization profile across interfaces, mean magnetization vector in the film plane, and magnetic domain distribution (dispersion) for varying external magnetic fields. In the case of periodic multilayers stacked in the direction normal to the film plane, with PNR methods, one can distinguish between ferromagnetic, antiferromagnetic or non-collinear correlation between consecutive magnetic layers. Off-specular diffuse scattering adds information on nuclear and magnetic roughness, and in particular, on magnetic domain fluctuations in the longitudinal and transverse direction. All this can experimentally be probed and theoretically be analyzed; only a few aspects of this information could be discussed in this contribution in any more detail. Specifically, we have described polarized neutron reflectivity on a thin magnetic iron film and the information that can be gained with a one-dimensional polarization analysis in comparison to an experimental situation without polarization analysis, or one with unpolarized neutrons. Considering laterally patterned magnetic films instead of flat samples, it is important to take into account the spatial orientation of the coherence volume. In the case of stripe arrays, two main orientations with respect to the neutron polarization and the scattering plane are to be probed: stripes parallel and perpendicular to the scattering plane. The perpendicular orientation reveals the periodicity of the stripe pattern. In the parallel orientation, the stripe edge roughness and the flux distribution in the interspatial region can be probed by the off-specular diffuse scattering.

More generally, if the stripes were arranged randomly, they could be taken as a rough magnetic film, featuring randomly separated islands with flats in between. This topology would give rise to off-specular diffuse scattering close to the total reflection regime without featuring Bragg reflections. The other extreme, a perfect periodic arrangement of stripes would act as diffraction grating on neutrons and causes lateral Bragg reflections. Edge roughness, waviness of the stripes, variations of stripe separation and stripe thickness, magnetic flux distribution in and between the stripes are all topics that can be addressed via specular polarized neutron reflectivity and off-specular Bragg and diffuse scattering. Clearly, periodic stripe arrays is the most basic realization of a lateral periodic structure. Any other lateral pattern, such as artificial spin ice [24,25], Penrose tiles, or chiral domain wall patterns including skyrmions lattices [26] may be thought of and can be studied by the methods discussed here, albeit with increasing complexity.

Finally we would like to emphasize that PNR on thin magnetic films and multilayers is an indispensable, highly reliable, and a straight forward tool for gaining essential information on spintronic and magnonic materials. It is complementary to X-ray resonant magnetic scattering (XRMS) in the hard and soft X-ray regime. Nowadays, XRMS is more frequently available for experimentalists because of the higher number of synchrotron radiation sources compared to neutron sources. On the other hand, the analysis of XRMS spectra is more intricate than the analysis of PNR scans because the latter method is based on resonance absorption of circular polarized X-rays, which cannot be treated by the more simple Born approximation or distorted wave Born approximation. For more information on the fundamentals of PNR and XRMS we refer to a number of excellent reviews articles [7,16,19,27,28,29,30,31].
